# ALK-positive Spitz nevus of the nasal dorsum in a child: a case report and literature review

**DOI:** 10.3389/fonc.2026.1845068

**Published:** 2026-07-02

**Authors:** Wang Juan, Zhifang Zhai

**Affiliations:** Southwest Hospital, Army Medical University, Chongqing, China

**Keywords:** ALK positive, fusion gene, malignant melanoma, prognosis, Spitz mole

## Abstract

This article reports a case of Spitz nevus with ALK positive nasal cavity in a 3-year-old and 5-month-old female child. The clinical manifestation is red patches on the nose of the child, gradually increasing in size, without itching or pain. Histopathological examination showed a large proliferation of spindle shaped cells in the dermis, without any evidence of nuclear fission. Spindle shaped cells show positive reactions to markers such as S100, SOX10, ALK, P16, MelanA, etc. Pathological diagnosis of Spitz nevus with ALK positivity. Spitz nevus is a benign proliferative lesion of melanocytes, commonly found in children and adolescents, with specific clinical and pathological features. ALK gene fusion is more common in Spitz nevi and helps in differential diagnosis from malignant melanoma. Most ALK positive Spitz nevi exhibit benign biological behavior, but close follow-up is still necessary to assess potential risks. Future research should further explore the relationship between fusion genes and the biological behavior of Spitz tumors.

## Case report

**Figure 1:**
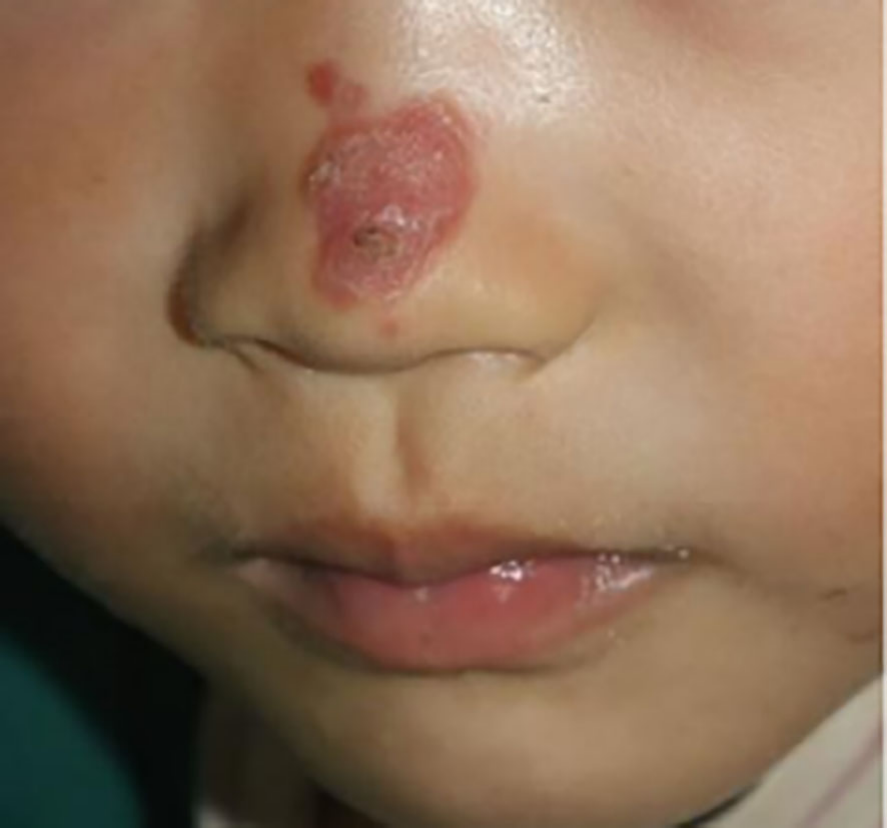
A red patch measuring approximately 2.5 cm × 1.5 cm on the nasal dorsum, slightly elevated, with clear borders and an irregular shape. The surface shows slight scaling and crusting, without blisters, ulceration, or exudation. Two millet-sized satellite lesions are observed around the main lesion.

**Figure 2:**
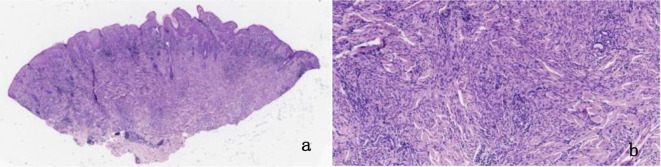
Histopathological features include hyperkeratosis with parakeratosis, crust formation, acanthosis, and elongation of rete ridges. The dermis shows a proliferation of spindle cells without pigment or mitotic figures. The lesion is located throughout the dermis layer, with symmetry, maturity, and no cellular abnormalities, no appendages affected. [**(a)** HE ×40, **(b)** HE ×200].

**Figure 3:**
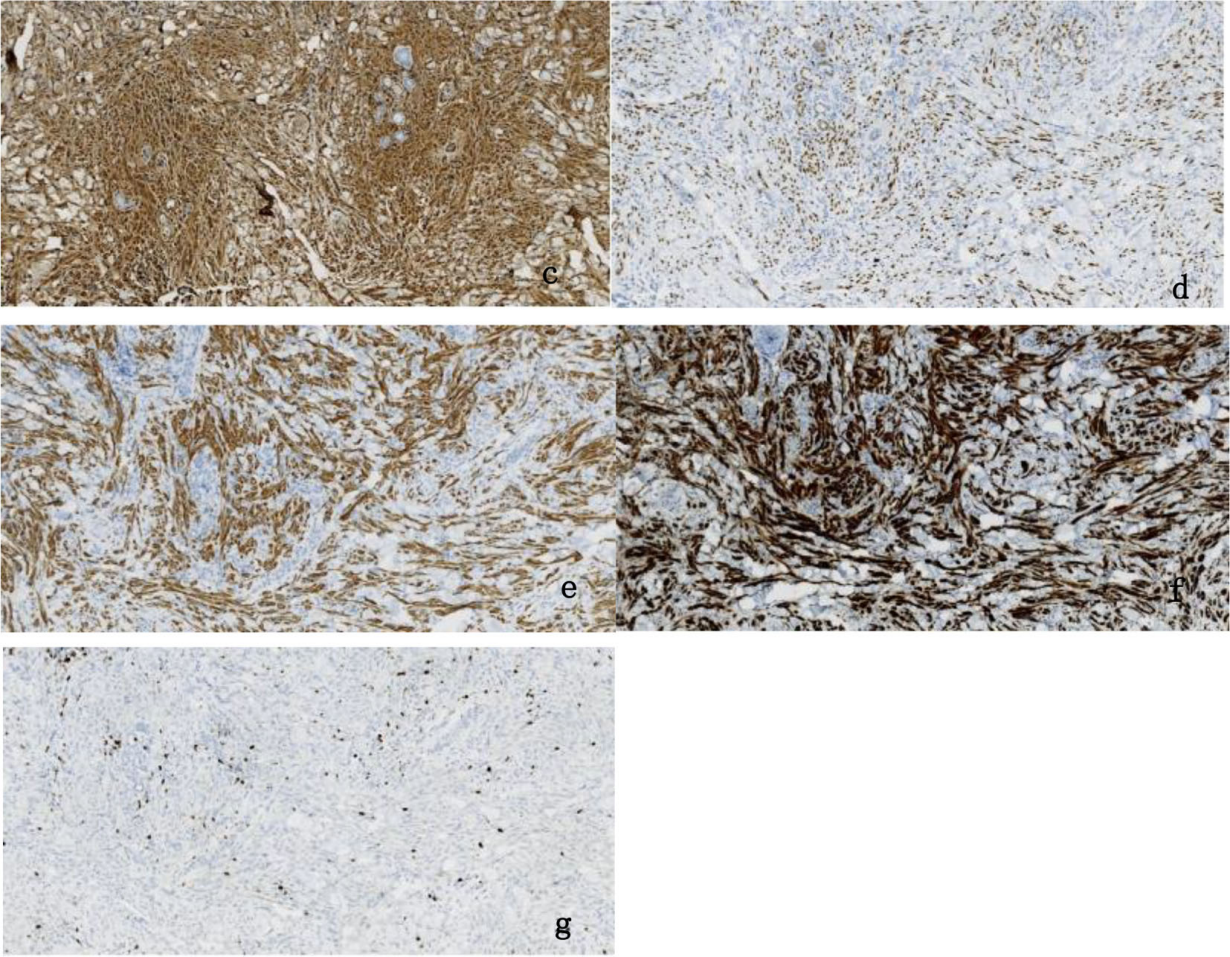
Immunohistochemical analysis shows positive expression of S100, SOX10, ALK, P16, and MelanA in spindle cells (ALK positivity cytoplasmic and membranous) **(c–f)**, and a Ki67 proliferation index of approximately 2% in hotspots **(g)** (×200).

The patient was a 3-year-and-5-month-old girl who presented with an asymptomatic erythematous patch on the nasal dorsum that had gradually enlarged over six months, without pain or itching. Dermatological examination revealed a well-defined, slightly elevated red patch with pear-shaped and the size of 2.5 cm × 1.5 cm on the nasal dorsum. The surface showed slight scaling and crusting, without blisters, ulceration, or exudation. Two satellite foci were observed in the upper right side of the lesion (**Figure 1**). Histopathology revealed hyperkeratosis with parakeratosis, crust formation, acanthosis, and elongation of rete ridges, and a proliferation of spindle cells without pigment or mitotic figures were found in the dermis. The lesion is located throughout the dermis layer, with symmetry, maturity, and no cellular abnormalities, no appendages affected. (**Figure 2**). Immunohistochemical examination revealed positive expression of S100, SOX10, P16, MelanA and diffuse strongly positive ALK for all spindle cells(ALK positivity cytoplasmic and membranous), with partially positive for CD45, Bcl2, and HMB45, and focal positive for CyclinD1.HMB45 is slightly positive in the superficial dermis. Proliferation index of Ki67 was approximately 2% in hotspots and PRAME, CK, and CD31 were negative (**Figure 3**). The diagnosis of ALK-positive Spitz nevus was established. Although most Spitz nevi exhibit benign biological behavior, our particular case presented with a large lesion (2.5 cm) accompanied by satellite foci and gradual enlargement, histopathology demonstrated deep nevus cell infiltration. Therefore, complete surgical excision was recommended. The patient subsequently underwent surgical treatment in the Department of Plastic and Aesthetic Surgery at our hospital, and the postoperative pathological diagnosis was consistent with the preoperative pathological biopsy. There was no recurrence after more than one year of postoperative follow-up. The patient remains under close clinical follow-up because of the lesion’s atypical clinical features.

## Discussion and literature review

Spitz nevus is a benign melanocytic proliferative lesion first described by Sophie Spitz in 1948. Spitz nevi are relatively rare in children with melanocytic nevi and typically present as solitary lesions, predominantly in children and adolescents. About 80% of cases occur in patients under 20 years of age. Spitz nevi more commonly occur on face, head and neck lower extremities. Spitz nevi exhibit variable morphology. Spitz nevus is a benign melanocytic tumor. The vast majority of Spitz nevi follow a stable, non-aggressive course without progression. Most lesions remain unchanged or may slowly regress over time. Histopathological features showed that Characterized by large epithelioid or spindle melanocytes with maturation gradient. Mitotic activity (if present) is typically low and confined to superficial dermis. Approximately 50% harbor kinase fusions (e.g., *ALK, ROS1, NTRK), which are not predictive of malignant behavior. Complete excision (when performed) shows >95% cure rate. Even with conservative management, <1% show atypical changes requiring re-intervention. No increased melanoma risk in patients with classic histology. Rare “atypical” variants may require extended follow-up (5+ years) (1).

Although benign, histologically it can be challenging to distinguish from spitzoid melanoma. Spitzoid tumor cells exhibit epithelioid or spindle morphology, arranged in nests, cords, or scattered patterns from the dermoepidermal junction to the deep dermis. In contrast, Spitzoid melanoma demonstrates loss of nested architecture and maturation, with extensive pagetoid spread. The tumor cells exhibit marked cytologic atypia, frequent mitoses, large and prominent nucleoli, and may show ulceration or tumoral necrosis. A band-like inflammatory infiltrate is often present.

Studies have shown that Spitz tumors harbor various gene fusions, including ALK gene fusions, which are associated with specific clinicopathological features. Diffuse ALK immunoreactivity strongly suggests the presence of ALK rearrangement, although molecular confirmation is preferable (2, 3). In one study, the authors analyzed the correlation between histopathological features and gene fusions in 17 Spitz tumors with ALK fusions (5 Spitz nevi and 12 atypical Spitz tumors). All cases were confirmed to have ALK gene rearrangements by FISH, with 11 cases showing TMP3-ALK fusions (5 Spitz nevi and 6 atypical Spitz tumors) and 6 cases showing DCTN1-ALK fusions (all atypical Spitz tumors) (4). The clinicopathological histochemical features of ALK-positive Spitz nevi are summarized in the following table (**Table 1**).

**Table 1:** The clinical and pathologic characteristics of all nearly 10 years reported cases of Spitz nevus.

Cases	Ethnicity	Age (year)/duration	Sex	Diseased parts	Lesion appearance	Pathologic features	Histochemistry features	Therapy response
Brown RA et al (5)	American	3/1year	Female	lower lip	Exophytic papule on vermilion border	Complex melanocyte hyperplasia with well-defined borders, epithelioid melanocytes in the epidermis, and spindle cells in the dermis in a cluster-like arrangement with Schwann-like structures. Melanocytes extensively infiltrate perineural and intraneural areas.	Melanocytes expressed ALK, S100 and Melan-A, BRAFV600E was negative, and the Ki-67 proliferation index was <5%. FISH testing showed no abnormalities.	No recurrence 3 months after surgery.
Ren J et al (6)	Chinese	4-47/1-36months	2 Males,11 Females.	5 cases of head and face, 3 cases of calves, and 1 case of ankles, thighs, knees, elbows and buttocks.	Most present with papular nodules, and plaques, pink, brown-red, or gray-black	Most of the lesions were predominantly exophytic (9/13), and more than half of the cases had no obvious melaninosis, and the intraepidermal junction components were dominated by melanocyte nest distribution, and no kamino bodies were found. The basal configuration of the tumor is mainly wedge-shaped (5/13) and flat (7/13). The morphology of tumor cells was a mixture of epithelioid cells and spindle cells in 8 cases, a single spindle cell morphology in 5 cases, all diseased cells showed a plexiform and/or cross-bundled growth pattern, and 3 cases had small nerve encirclement.	Immunohistochemistry of 13 cases of ALK showed strong cytoplasmic diffuse strength of tumor cells, and 7 of them were confirmed by FISH testing.	No recurrence 7–21 months after surgery.
Minowa T et al (7)	Japanese	33/1year	Female	the left forearm	a 9-mm, reddish-papule	Diffusely distributed tumor cell nests with a bulbous border in the dermis and the subcutaneous fat. Rosette-like structures, composed of epithelioid cells, were observed throughout the lesion. There were no tumor cells in the epidermis. The tumor cells lacked maturation. Neither Kamino bodies norfusiform melanocytes with a fascicular growth pattern were observed. Scant mitoses were observed.	The tumor cells were positive for Melan-A, MITF, and ALK.RNA sequencing showed high expression of ALK exons, but no fusion genes such as ALK/NTRK1 or BRAF/NRAS mutations were detected.	No recurrence 9 months after surgery.
Rand AJ et al (8)	American	9/4;7/4year	Male;Female	(1)on the anterior left thigh;(2)on the anterior right ankle	Pedunculated; (1)14 mm irregularly pigmented(2)12mm lightly pigmented	(1)Nests of spindled to polygonal melanocytes with large nuclei, amphiphilic fibrillar cytoplasm and prominent clefting;(2)Low power examination revealed a polypoid proliferation of cells forming tightly packed nests in the dermis. At higher magnification, there was a broad junctional comp.	(1)Strong diffuse cytoplasmic positivity for ALK. FISH confirmed ALK rearrangement. BAP1 was retained and BRAF V600E was negative by IHC;(2)The tumor was negative for a TERT promoter mutation.	9 months of interferon therapy and is doing well ;doing well (1 month after surgery).
Kiuru M et al (9)	American	2 to 59, with a mean age of 21 years/	None	None	None	the growth pattern of intersecting fascicles of amelanotic fusiform melanocytes and lacking epithelioid cell morphology.	28 tumors (28 of 105 or 26.7%) were immunopositive for ALK. NTRK1 expression was observed in 20 tumors(20 of 105 or 19.0%).	None
Fujimoto M et al (10)	Japanese	40/6 months	Female	right forearm	red polypoid lesion (0.8 cm in size)	The tumor was situated asymmetrically in the dermis, and a junctional component was not observed. The tumor was composed of fascicular nests of nonpigmented spindle and epithelioid tumor cells with prominent nucleoli and clefts between the tumor cells.	MelanA, S100, positive, ALK Negative; ALK split FISH analysis demonstrated ALK rearrangement.	No recurrence or metastasis for 9-year after surgery.

Most of these previously reported cases come from the United States, Japan, and China. The age of onset ranges from 2 years old to 59 years old and has no distinctive features. There is no gender difference. No characteristic features of any part. The main manifestations of skin lesions are papules, nodules, and plaques, which are red, pink, brownish red, and black. All cases showed ALK positivity or rearrangement. Almost all cases have a good prognosis after surgical resection. Intersecting fascicular growth of fusiform melanocytes was seen in all but one ALK-positive tumor. There was a trend toward ALK-positive tumors being amelanotic and lacking epithelioid cell morphology. This study confirms that although not specific, the growth pattern of intersecting fascicles of amelanotic fusiform melanocytes is strongly associated with ALK expression (9). Some ALK-positive compound Spitz nevus with extensive perineural and intraneural neurotropism. Some cases show the transformation of tumors produced by an activating kinase fusion gene (ALK) through secondary genetic changes including loss of tumor suppressor activity (CDKN2A). Long-term follow up will be important to further define the behavior of these unique Spitz tumors (8). The majority of these tumors have an excellent prognosis (6).

ALK-positive Spitz nevi typically exhibit a fascicular arrangement of spindle cells in the dermis, often lacking pigment but occasionally containing minimal pigment. Immunohistochemically, these melanocytic cells express ALK (11). Additionally, Spitz nevi often express other melanocytic markers such as Melan-A, SOX10, and S100, while HMB-45 expression shows a shallow layer pattern. The primary differential diagnosis for Spitz nevus is malignant melanoma. Unlike Spitz nevi, malignant melanoma exhibits disordered growth patterns, irregular borders, and mitotic figures, including atypical mitoses, in the deep dermis. Full-thickness HMB45 expression is a diagnostic indicator of malignant melanoma. In contrast, Spitz nevi may show occasional single nevus cells in the upper epidermis but generally lack pagetoid spread. In this case, the absence of mitotic figures, strong ALK expression in spindle cells, positive expression of Melan-A, SOX10, S100, and P16, shallow layer HMB45 positivity, negative PRAME, and low Ki67 proliferation index, combining clinical and prognostic factors supported the diagnosis of a benign ALK-positive Spitz nevus (3, 12). ALK immunostaining alone is not equivalent to confirmation of ALK fusion/rearrangement. Confirmation of subsequent ALK fusion/rearrangement was not conducted due to economic reasons of the patient. However, we diagnosis based on the patient’s clinical presentation, histopathology, and immunohistochemistry.

Literature reports indicate that most ALK-positive Spitz nevi and atypical Spitz tumors exhibit benign biological behavior, with no recurrence or sentinel lymph node metastasis. However, close follow-up and monitoring are necessary for atypical Spitz tumors with malignant potential. The preferred treatment for ALK positive Spitz nevi is complete surgical resection, followed closely by postoperative follow-up (13).

Our case report describes an ALK-positive Spitz nevus on the nose and reviews the literature on the correlation between ALK gene fusions and Spitz nevi. ALK-positive Spitz nevi exhibit specific morphological and immunohistochemical features that aid in differentiating them from malignant melanoma. Further research is needed to explore the biological behavior and clinical significance of gene fusions in Spitz tumors. ALK-positive Spitz nevi are benign melanocytic tumors with distinct clinicopathological features. Through literature review, we understand that these tumors are more common in young patients and exhibit specific histopathological and immunohistochemical characteristics. Differential diagnosis requires distinguishing them from malignant melanoma and other melanocytic tumors. Although most ALK-positive Spitz nevi exhibit benign behavior, close follow-up and monitoring are essential to assess potential risks. With advancements in molecular biology and histopathological techniques, we will be able to diagnose and treat these tumors more accurately in the future (14).

## Data Availability

The original contributions presented in the study are included in the article/supplementary material. Further inquiries can be directed to the corresponding authors.
